# The cellular signature of urinary immune cells in Lupus nephritis: new insights into potential biomarkers

**DOI:** 10.1186/s13075-015-0600-y

**Published:** 2015-04-03

**Authors:** Katharina Kopetschke, Jan Klocke, Anna-Sophie Grießbach, Jens Y Humrich, Robert Biesen, Duska Dragun, Gerd-Rüdiger Burmester, Philipp Enghard, Gabriela Riemekasten

**Affiliations:** Department of Rheumatology and Clinical Immunology, Charité Universitätsmedizin Berlin, Berlin, Germany; Department of Rheumatology, Universitätsklinikum Schleswig-Holstein, Lübeck, Germany

## Abstract

**Introduction:**

Urinary T cells represent a reliable noninvasive biomarker for proliferative Lupus nephritis (LN). Little is known about the presence of T cell subsets, B cells and macrophages in the urine although they may further improve the validity of urinary cellular biomarkers for LN.

**Methods:**

We analyzed contemporaneous blood and urine samples of patients with active LN (n = 19), other Systemic Lupus Erythematosus (SLE) patients (n = 79) and urine samples of patients with diabetic nephropathy (DN; n = 14) and anti-neutrophil cytoplasmatic antibody (ANCA) associated vasculitis (AAV; n = 11) by flow cytometry.

**Results:**

Numbers of urinary T cells, B cells and macrophages correlated with disease activity and were significantly higher in the active LN group. Urinary T cells showed excellent distinction of patients with active LN, CD8+ T cells (AUC of ROC = 1.000) and CD4+ T cells (AUC = 0.9969) alike. CD19+ B cells (AUC = 0.7823) and CD14+ macrophages (AUC = 0.9066), as well as the clinical standard proteinuria (AUC = 0.9201), failed to reach these high standards. Patients with DN or AAV also showed increased urinary cell counts, although the CD4/CD8-ratio was significantly lower in SLE compared to in DN (p = 0.0006). Urinary CD4+ T cells of active LN patients proved to be mainly of effector memory phenotype and expressed significantly more CD40L and ki67 than corresponding blood cells. Urinary Treg counts correlated with disease activity.

**Conclusions:**

Despite of detectable urinary cell counts for B cells and macrophages, T cells remain the best urinary cellular biomarker for LN. A low CD4/CD8-ratio seems to be characteristic for LN.

**Electronic supplementary material:**

The online version of this article (doi:10.1186/s13075-015-0600-y) contains supplementary material, which is available to authorized users.

## Introduction

Lupus nephritis (LN) is one of the most common manifestations of systemic lupus erythematosus (SLE) [1]. Although therapy has improved over the years, LN is still one of the most threatening complications implying the hazard of terminal renal failure and increased mortality [2]. Current care of patients with LN may further be improved by establishing new biomarkers for diagnosis and treatment monitoring, facilitating early diagnosis and helping avoid over- and under-treatment [3,4].

Kidney biopsy is usually applied to diagnose LN in SLE patients with a combination of systemic disease activity and abnormally elevated urinary markers, such as proteinuria [5]. The potential inaccuracy of the established urinary markers and the risk of invasive biopsy [6] led to the search for alternative biomarkers. Although both serum and urine have been examined for viable markers, urinary compounds are generally considered to show a better reflection of renal inflammation and irreversible kidney damage [7-9].

In a recent study we were able to show that urine samples of patients with acute proliferative LN contain high amounts of CD3 + CD4+ T cells which can be assessed by flow cytometric analysis [10]. The T cell count can be used as a biomarker for proliferative LN among SLE patients [10,11]. The urinary cells are also a phenotypical correlate of the kidney’s interstitial infiltration [12,13], which is a common element of LN and correlates closely with disease activity and kidney damage [14-16]. This resemblance leads to the assumption that the urinary cells originate from the inflamed kidney rather than peripheral blood. Cells in the infiltrate are comprised mainly of T cells but also of macrophages, B cells and plasma cells [16-18]. Based on our hypothesis, other cell types besides CD3 + CD4+ T cells should also be detectable in the urine of LN patients and may be used as biomarkers.

The urine - and urinary cells in particular – can possibly be used to noninvasively explore the cellular components of the inflammatory renal environment. In addition, assessment of urinary cells may yield predictive markers for the patients’ outcome, therapy response or future nephritis flares. We and others previously demonstrated that urinary T cells are also found in other nephropathies with inflammatory infiltration, such as diabetic nephropathy (DN) or anti-neutrophil cytoplasmatic antibody (ANCA)-associated vasculitis (AAV) [10,19]. As yet there is no evidence on whether there are any differences between diseases in the occurrence or composition of urinary cells.

In the present study we analyzed the urinary cellular profile of patients with SLE, DN and AAV for T cells and their subsets, B cells and macrophages in order to obrain a thorough view of urinary cells in SLE, and further refine their diagnostic value as biomarkers for LN.

## Methods

### Ethics approval

Ethics approval was obtained from the *Ethikkomission Charité*.

### Patients

In the period between April 2009 and March 2013 a total of 123 patients were recruited for this study (for detailed patient characteristics see Table 1). We collected and analyzed samples from 98 patients with SLE, 14 patients with DN and 11 patients with AAV.

**Table 1:** **Patient characteristics**

	**SLE, active renal disease**	**SLE, non-active renal disease**	**Diabetic nepharopathy**	**ANCA associated vasculitis**
Number	19	79	74	11
Female/male, number	17/2	71/8	6/8	4/7
Age, years	31 (19 to 60)	44 (21 to 72)	58 (27 to 93)	63 (38 to 78)
SLEDAI	14 (11 to 23)	2 (0 to 10)	na	na
BVAS	na	na	na	5 (0 to 16)
Immunosuppresive treatment	19 × Pred 7 × Cyc, 1 × Aza 13 × HCQ, 2 × MMF	62 × Pred, 20 × Aza, 37 × HcQ, 15 × MMF, 3 × MTX, 3 xBelim, 1 × Flebo	1 × Rtx	9 × Pred, 1 × Cyc, 6 × Aza, 2 x Rtx
Prednisolone dose, mg, median (IQR)	40 (30 to 50)	5.0 (5.0 to 7.5)	-	5.0 (2.5 to 5.0)

Values are mean (range) unless stated otherwise. Aza, azathioprine; Belim, belimumab; BVAS, Birmingham Vasculitis Activity Score; Cyc, pulse cyclophosphamide; HCQ, hydroxychloroquine; Flebo, flebogamma; MMF, mycophenolate mofetil; MTX, methotrexate; na, not applicable; Pred, prednisolone; Rtx, rituximab; SLE, systemic lupus erythematosus; SLEDAI, systemic lupus erythematosus disease activity index.

We divided the SLE population into a group with active renal involvement (n = 19) and a group with non-active renal involvement (n = 79). The systemic lupus erythematosus Disease activity index (SLEDAI) was calculated for all patients. Active renal involvement was defined by high overall disease activity (SLEDAI ≥10) and a current kidney biopsy (not older than 4 weeks) showing LN (n = 14). In the absence of a biopsy, active renal involvement was defined by high disease activity (SLEDAI ≥10) and at least two elements of the renal SLEDAI (n = 5). All examined biopsies showed class IV (n = 10) or class IV + V (n = 3) except one case of pauci immune glomerulonephritis (see Additional file 1: Table S1).

Clinical follow-up data 6 months after the inclusion in the study were retrospectively retrieved from the medical files and were available for patients with initially active LN and 20 SLE patients who initially had no disease or had mildly active disease (SLEDAI <10). If 6-month follow-up data were not available, follow-up data collected 5 or 7 months after the urine analysis were accepted as a substitute. Remission was defined as a reduction in proteinuria by at least 50% and improvement or stabilization of creatinine 6 months after initiation of therapy for proliferative LN; refractory disease was defined as failure to achieve remission. In our cohort of eight patients with active LN, five achieved remission. Development of a new renal flare was defined as an increase in proteinuria or worsening creatinine attributed to SLE activity by the treating physician. In our cohort two patients developed a new renal flare.

Datasets of 71 SLE patients were included in the analysis of urinary cells, and the T cell subset analysis was performed in 53 SLE patients. Due to limited urinary cell numbers not all tests could be performed in every patient. No patients were excluded after analysis.

Our AAV cohort consisted of eight cases of granulomatosis with polyangiitis (formerly known as Wegener’s granulomatosis) and three patients with perinuclear ANCA-associated disease. All patients had AAV-related renal involvement. AAV disease activity was measured by the Birmingham vasculitis activity score (BVAS).

Samples were collected from patients of the Department of Rheumatology and Clinical Immunology and Nephrology, Charité University Hospital, Berlin, Germany. The ethics committee of Charité University Hospital (Charité EA1/034/10) approved the study. Informed consent was obtained from all patients before participation.

### Sample preparation and flow cytometry

Urine samples were collected and immediately centrifuged. Pellets were washed with PBS/ BSA. The median sample size was 100 ml urine. To assure a high percentage of living urinary cells during measurements, only fresh urine was analyzed, while samples older than 6 h were discarded. Peripheral blood mononuclear cells were acquired by incubating fresh blood samples for 20 minutes at 4°C with erythrocyte lysis buffer and washing with PBS/BSA. Cells were stained with CD3-PE, CD4-PE, CD4-FITC, CD8-PE/Cy7, CD14-PerCP/Cy5.5, CD25-FITC, CD28-PerCP/Cy5.5, CD40L-FITC, CD45RO-APC, CD127-APC, CCR7-FITC and FoxP3-Alexa488 (all Biolegend, San Diego, CA, USA), CD3-APC/Vio770 and CD25-APC (both Miltenyi Biotec GmbH, Bergisch Gladbach, Germany), CD4-Cy5, CD8-FITC, CD14-FITC and CD19-Cy5 (all DRFZ) and CD127-eFluor (eBioscience, San Diego, CA, USA).

To block unspecific binding, cells were stained in PBS/BSA containing 10% human IgG (Flebogamma; Grifols, Langen, Germany); to exclude dead cells, either propidium iodide (Sigma-Aldrich, Steinheim, Germany) or diamidino phenylindole (Sigma-Aldrich, Steinheim, Germany) was added immediately before flow cytometry. In case of fixation, exclusion of dead cells was achieved by a Dead Cell discrimination kit (Miltenyi Biotec GmbH, Bergisch Gladbach, Germany). To calculate cell numbers, defined sample sizes were acquired and the number of cells normalized as cells/dl of the initial urine sample. The samples were measured using a Calibur flow cytometer (BD Pharmingen, Heidelberg, Germany) and a MACS Quant Analyzer (Miltenyi Biotec GmbH, Bergisch Gladbach, Germany). Datasets were analyzed using Flowjo Software (Tree Star, Ashland, OR, USA).

### Routine laboratory values

Routine laboratory values for serum creatinine and proteinuria were additionally gathered. Creatinine was measured by the Jaffe reaction, 24-h urinary protein excretion using a turbimetric assay.

### Statistical analysis

Medians, Mann–Whitney tests, Wilcoxon matched pairs tests, Spearman correlation, and receiver operator characteristic (ROC) curves were calculated using GraphPad Prism 5 (GraphPad Software, San Diego, CA, USA).

## Results

### Patients with active LN had elevated amounts of urinary cells

**Figure 1:**
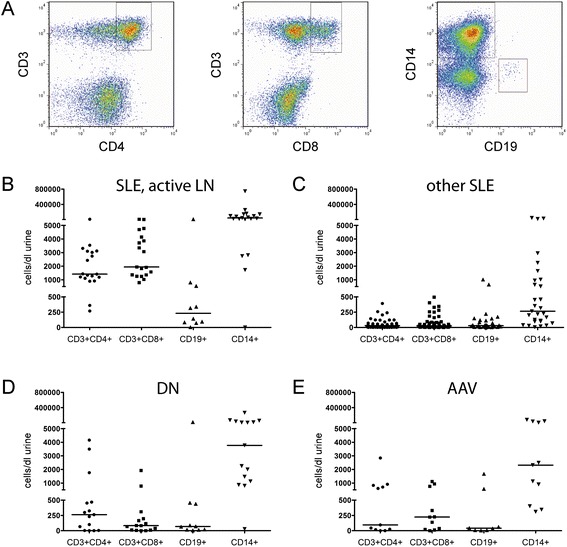
**Urinary cells in systemic lupus erythematosus (SLE) patients and in patients with different nephropathies. (A)** Examplary dot-plots of the flowcytometric analysis of urinary T cells, B cells and monocytes in a patient with acute proliferative lupus nephritis (LN). **(B**-**E)** Urinary cells in patients with acute proliferative LN **(B)**, other SLE patients **(C)**, patients with diabetic nephropathy (DN) **(D)** and patients with ANCA-associated vasculitis (AAV) **(E)**.

Urine samples from patients with high disease activity (SLEDAI ≥10) and recent biopsy-proven LN (n = 14) or high renal disease activity (SLEDAI ≥10 and renal SLEDAI ≥8, n = 5) had high numbers of both CD4+ (median 1,415 cells/dl urine) and CD8+ T cells (median 1,911 cells/dl urine). Even higher numbers of urinary CD14+ macrophages were observed with a median of 33,808 cells/dl urine (n = 19). In comparison, CD19+ B cells (median 231cells/dl urine) were only moderately increased in the urine of active patients (n = 10) (Figure 1A and B).

In SLE patients without active renal involvement only low numbers of T cells were detected (median 29 CD4+ and 25 CD8+ T cells/dl urine, n = 55). With a median of 29 cells/dl urine, CD19+ B cell counts were similar to T cell counts in these patients (n = 29). However we frequently observed considerable amounts of CD14+ macrophages in the urine of these patients, (median 264 cells/dl urine, n = 29) (Figure 1C). Differences in urinary cell counts between patients with active and non-active SLE were highly significant for CD3 + CD4+, CD3 + CD8+ and CD14+ cells (*P* <0.0001) and to a lesser degree also for CD19+ cells (*P* = 0.0137). The disease activity (SLEDAI) correlated with the urinary cell counts of CD3 + CD4+ (*r* = 0.7363, *P* <0.0001), CD3 + CD8+ (*r* = 0.7641, *P* <0.0001), CD14+ (*r* = 0.5715, *P* <0.0001) and CD19+ cells (*r* = 0.4636, *P* <0.0030).

In DN patients, a median of 262 CD4+ and 82 CD8+ urinary T cells were detected, which was slightly higher than CD19+ B cell counts (median 69/dl) and considerably lower than CD14+ macrophage numbers (median 3,770/dl) (Figure 1D). Patients with AAV had variable urinary lymphocyte counts, while urinary macrophages were detectable in all samples (Figure 1E). Cell counts did not correlate with the BVAS (data not shown).

### CD4/CD8-ratio was shifted towards CD8 in the urine of SLE patients and varied between different renal diseases

**Figure 2:**
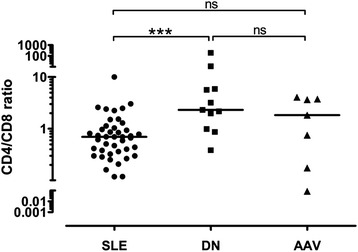
**Urinary CD4/CD8-ratio in various renal diseases.** All patients with CD3+ cell counts >100/dl were included. SLE, systemic lupus erythematosus; DN, diabetic nephropathy; AAV, ANCA-associated vasculitis.

In order to determine the relations between urinary CD4+ and CD8+ cell counts in different renal diseases, a CD4/CD8 ratio was calculated for urine samples of patients with at least 100 CD3+ cells/dl urine. The examined cohorts consisted of 38 SLE patients, 11 patients with DN and 7 patients with AAV (Figure 2).

Whereas SLE patients typically had slightly higher urinary CD8+ than CD4+ T cell counts (median 0.696 urinary CD4/CD8-ratio) regardless of disease activity or known renal involvement, patients with severe DN showed reciprocal proportions in most cases (median 2.306 urinary CD4/CD8-ratio). The difference between urinary CD4/CD8-ratios in patients with SLE and DN was highly significant (*P* = 0.0006). Patients with AAV had a high range of CD4/CD8-ratios (median 1.826 CD4/CD8-ratio, ranging from 0.085 to 4.067), which did not correlate with the BVAS. Comparing the ratios of AAV patients with SLE (*P* = 0.2591) or DN patients (*P* = 0.2391) did not lead to any significant results. Other formed ratios, CD3/CD14 and CD3/CD19, did not yield any significant differences between diseases, either (data not shown).

### Subtyping of urinary CD4+ T cells in LN

**Figure 3:**
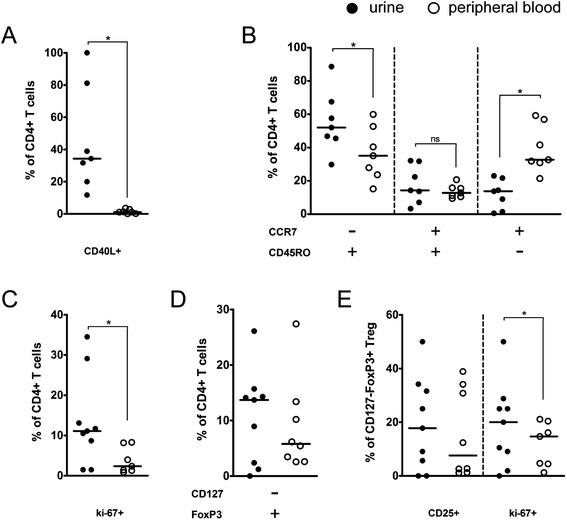
**Subtyping of CD4+ T cells in systemic lupus erythematosus (SLE) patients in urine and peripheral blood.** Filled circles = urine; open circles = peripheral blood. **(A)** Expression of CD40L on CD4+ T cells (n = 7). **(B)** Effector memory (CD45RO + CCR7-), central memory (CD45RO + CCR7+) and naïve (CD45RO-CCR7+) T helper cells (n = 7). **(C)** Proliferation rate as measured by ki-67 expression of CD4+ T cells (n = 10). **(D)** Regulatory (CD127-FoxP3+) CD4+ T cells (Treg) (n = 10). **(E)** Expression of CD25 and ki-67 in Treg (n = 10).

To evaluate the phenotype of urinary CD4+ T cells, urine and blood samples of 10 SLE patients with acute proliferative LN were analyzed for various T cell surface markers as well as intracellular markers ki-67 and FoxP3 (Figure 3). All patients had either recently undergone kidney biopsy (n = 9) or had high overall disease acitivity and renal disease activity (SLEDAI ≥10 and renal SLEDAI ≥8, n = 1). Total T cell subtype counts were also measured in additional urine samples from 43 SLE patients without acute renal involvement.

Urinary CD4+ T cells had a significantly higher expression of activation marker CD40L in comparison to the peripheral blood (*P* = 0.0156). The phenotype of urinary cells was shifted towards a higher percentage of effector memory type T helper cells (CD45RO + CCR7-) (*P* = 0.0156), while less naïve T cells (CD45RO-CCR7+) were found in the urine (*P* = 0.0156) when compared to peripheral blood. No differences between urine and blood CD4+ T cells were detected for the frequency of central memory (CD45RO + CCR7+), regulatory CD127-FoxP3+ T cells (Treg) and CD25+ Treg. The proliferation rate as measured by ki-67 of both overall CD4+ T cells and Treg was significantly increased in the urine (*P* = 0.0156 and 0.0313, respectively). Analysis of surface marker CD28 did not yield any significant differences between urine and blood for either CD4+ or CD8+ T cells (data not shown).

Correlation between the urinary cell counts of all measured patient samples with the SLEDAI was not significant for the urinary cell counts of CD40L+, naïve, effector memory or central memory T cells. Treg, however, correlated with the disease activity (n = 22, *r* = 0.5551, *P* <0.0073).

### Diagnostic value of urinary cells

**Figure 4:**
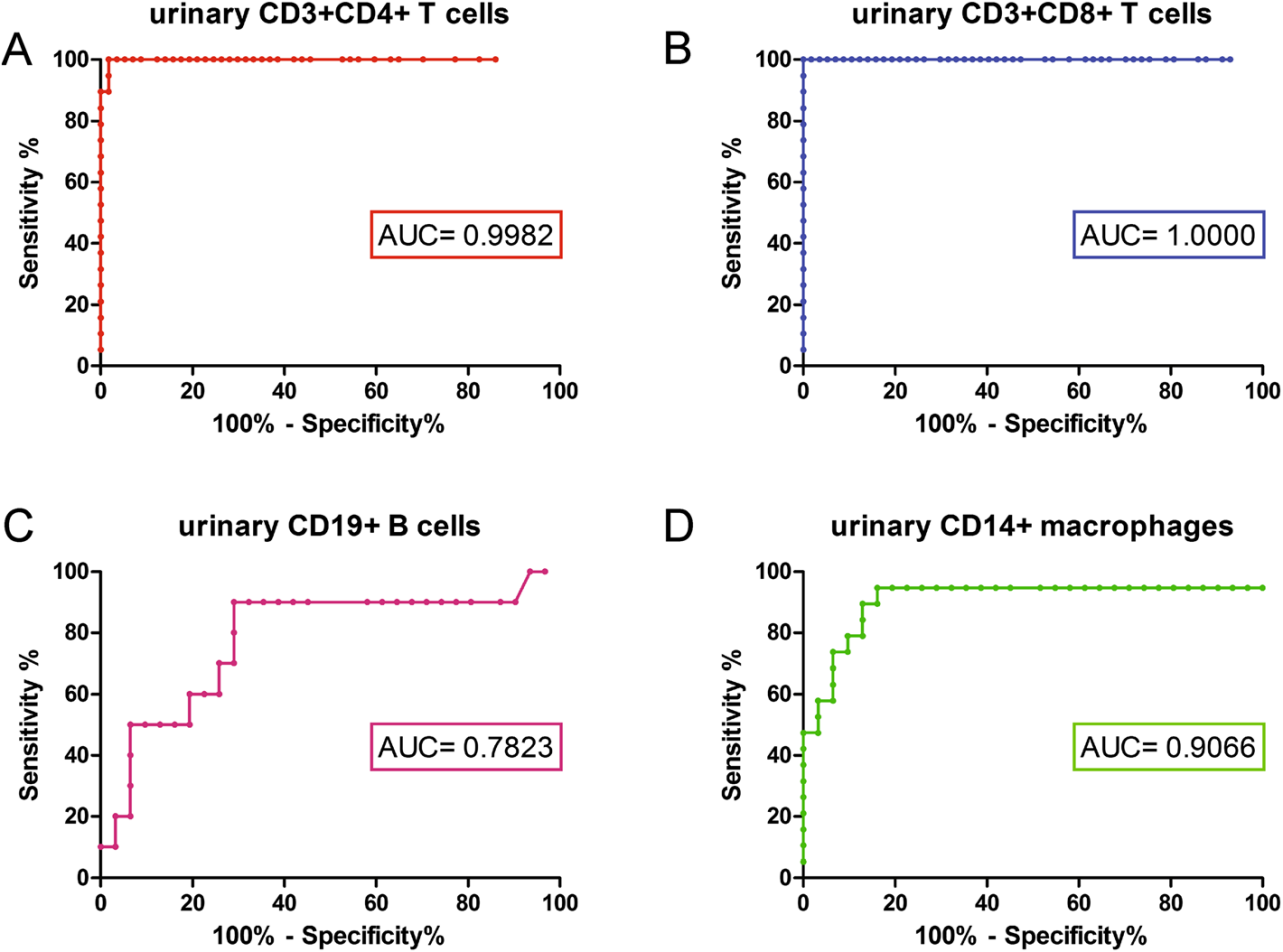
**Receiver operater characteristic (ROC) curve for diagnosing proliferative lupus nephritis (LN) among systemic lupus erythematosus (SLE) patients with various urinary cell types. (A)** CD3 + CD4+ T cells; **(B)** CD3 + CD8+ T cells; **(C)** CD19+ B cells; **(D)** CD14+ macrophages. AUC, area under the curve.

ROC curves were used to calculate and compare sensitivity and specificity of different urinary cell types and T cell subsets for identifying acute proliferative LN among SLE patients (Figure 4). For comparison, additional ROC calculations for approved clinical markers proteinuria and serum creatinine were made. The control group comprised other SLE patients with no active renal disease or no renal disease at all.

Of all tested markers (Table 2), CD8+ T cells yielded the highest diagnostic value with an area under the ROC curve (AUC) of 1.000. CD4+ T cells performed similarly well (AUC = 0.9969) and better than the established laboratory markers proteinuria (AUC = 0.9201) or serum creatinine (AUC = 0.6031). Urinary CD19+ B cells and CD14+ macrophages failed to reach these high standards, while subtyping of CD4+ T cells did not yield any improvement of the marker.

**Table 2:** **ROC for diagnosing proliferative LN among SLE patients with various urinary cell types as markers**

**Examined marker**	**Patients**	**Controls**	**Area under the ROC curve**	**Cutoff**	**Sensitivity**	**Specificity**
	**(n =)**	**(n =)**			**(%)**	**(%)**
**uCD3 + CD8+**	17	55	1.0000	>650 cells/dl	100	100
**uCD3 + CD4+**	19	55	0.9982	>270 cells/dl	100	98.25
**CCR7-CD45RO+**	6	34	0.9832	>300 cells/dl	100	94.12
**CCR7 + CD45RO+**	6	34	0.8172	>35 cells/dl	85.71	79.41
**CCR7 + CD45RO-**	6	34	0.7941	>45 cells/dl	100	52.94
**CD40l+**	6	34	0.9706	>100 cells/dl	100	70.59
**CD127-FoxP3+**	7	13	0.9017	>5 cells/dl	77.78	84.44
**Proteinuria**	16	45	0.9201	>620 mg/d	93.75	83.87
**uCD14+**	17	31	0.9066	>1700 cells/dl	94.74	83.87
**uCD19+**	10	31	0.7823	>75 cells/dl	90	70.97
**Creatinine**	19	55	0.6031	>1.1 mg/dl	47.37	79.17

Arranged according to area under the receiver operator characteristic (ROC) curve; cutoff determined by Youden’s index.

To evaluate whether a certain urinary immune cell composition predicts a clinical course, 6 (+/− 1)-month clinical follow-up data were retrieved from patients files. Clinical follow up was available for eight patients with initially active LN, five of whom achieved remission and three of whom were refractory. No significant differences in the initial urinary CD4+ T cell, CD8+ T cell, CD19+ B cell or CD14+ macrophage counts were observed. There was also no difference between refractory LN or response to therapy for regulatory CD4+ T cell or ki67 + CD4+ T cell counts. Among the 20 patients with non-active/mildly active SLE with 6-month clinical follow up, 18 patients showed no disease activity while one suffered severe, biopsy-proven LN class IV LN and another patient with known class V LN developed drastically increased proteinuria. Both patients had moderately elevated urinary T cell counts 7 months prior to the new renal flares (median 316 CD4+ T cells/dl and 268 CD8+ T cells/dl), while all other patients had only marginally detectable urinary T cells (median 13 CD4+ T cells/dl and 24 CD8+ T cells/dl).

## Discussion

This investigation provides information on the occurrence of several inflammatory urinary cell types in SLE and their relevance as a biomarker for proliferative LN. T lymphocytes were confirmed as the most reliable urinary biomarker for active LN, while urinary macrophages and B cells failed to emerge as diagnostic biomarkers. These urinary cells are not unique for LN but are also present in other inflammatory diseases like DN and AAV. However, the balance of urinary T cells seems to be shifted towards CD8+ T cells in LN.

Relevance of the local inflammatory infiltrating cells in the pathogenesis of LN is well-established [20]. Histology studies have reported that in LN the interstitial infiltration consists mainly of T cells and to a lesser extent of macrophages, B cells and plasmablasts/-cells [21]. Several authors describe a predominance of CD4+ T cells [17,21], while others report a majority of CD8+ T cells [22,23]. Solving this contradiction Winchester described different patterns of infiltration in LN, some predominated by CD4+ others by CD8+ T cells and also mixed CD4/CD8 infiltrations [24]. At present, the origin of immune cells in the urine is unknown and the relative contribution of periglomerular and peritubular infiltrates and large interstitial aggregates contribution to the urinary leukocytes is unclear. Nevertheless the cells in the urine seem to resemble the intra-renal cellular distribution, although macrophages outnumber other cell types in the urine.

Surprisingly high urinary macrophage counts were observed in several patients without clinical disease activity or known active nephritis. The significance of these cells is presently unknown, nevertheless it is tempting to speculate whether they reflect subclinical inflammation. Or, on the contrary, these macrophages may also exert anti-inflammatory effects, protecting the kidney against renal flares.

Based on our findings, a high percentage of urinary CD4+ T cells in SLE patients seemed to be of the effector memory phenotype. This matches the results of a recent report on LN [12] and similar observations that were made in acute AAV [19]. The higher activation (CD40L+) and proliferation (ki67+) of urinary CD4+ T cells stated in our results is plausible, given the inflammatory context and overall higher CD40L expression on T cells in proliferative LN [25]. In addition, a variable amount of Foxp3 + CD127- Treg was found in the urine (Figure 3) besides effector CD4+ T cells, which correlated with disease activity.

Urinary T cells have already been reported as an excellent biomarker to diagnose proliferative LN among SLE patients [10,11]. In the present study the urinary CD8+ T cell count was slightly superior to CD4+ T cells as a biomarker for active LN. Notably, both T cell types performed better in diagnosing LN than proteinuria or creatinine. Further sub phenotyping of urinary CD4+ T cells as performed in this study may be helpful for a better understanding of LN pathogenesis but does not improve T cells as a biomarker. Other urinary cell types did not hold up to the high standard of urinary T cells: while urinary B cell counts are in most cases too low in active LN patients to sufficiently distinguish them from other SLE patients, the high urinary macrophage count in some patients with non-active SLE leads to a large number of false positive results.

In a limited cohort we were able to probe whether certain immune cells in the urine may be predictive of a certain clinical course. Intriguingly, mildly elevated T cells seemed to potentially predict renal flares, which need to be analyzed in the future. Obviously our sample size and study design was inappropriate to make a definite conclusion but these observations are hypothesis-generating.

The occurrence of urinary cells in other diseases has already been reported previously [10,19,26,27]. Here we compared the urinary cell composition of LN with other nephropathies associated with an inflammatory infiltration: Concerning urinary cells, AAV seems to be a disease similar to LN. T cell counts correlating with disease activity have been reported for both renal manifestations [10,13,19] and no difference in the cellular composition in the urine was observed in our study. DN is increasingly considered an inflammatory disease [28]. In a recent study, Moon *et al*. indicated that intrarenal infiltration and activation of T cells in the interstitium is the main mechanism of kidney injury and showed a predominant CD4+ T cell renal infiltration in mice with induced diabetes [29]. In line with these reports we identified various amounts of urinary leucocytes in patients with DN. The assessment of urinary cells in DN patients revealed that overall cell counts are lower than in LN patients but proportions of T cells, B cells and macrophages are comparable. The ratio of urinary CD4+ and CD8+ T cells marks the only exception. Considering the decreased CD4/CD8-ratio in the peripheral blood of SLE patients [30], this finding is not surprising. However, these differences in the composition of urinary T cells might be helpful in the otherwise difficult urinary distinction between LN and other diseases: The CD4/CD8-ratio is a quotient usually used in the monitoring of T lymphocytes in HIV-positive patients or as a marker for diagnosing sarcoidosis via bronchial lavage [31,32]. However, when applied for urinary T cells, the ratio differs significantly between SLE and DN patients and reflects the differences in the respective intra-renal infiltrates. Other ratios, like the urinary CD14/CD3 ratio, which has been reported as helpful in differential diagnosis of glomerular diseases [26,27] did not yield any significant results. However, our data for both DN and AAV were restricted by low patient numbers and need to be confirmed by future studies.

## Conclusion

Our data present a comprehensive view of urinary cells in SLE. Urinary T cells, especially CD8+ T cells, maintain their leading role as cellular urinary biomarkers for proliferative LN, although both B cells and macrophages can be detected in the urine. In the near future, this non-invasive monitoring of urinary compounds might become a useful tool to facilitate clinical decisions on kidney biopsies and the treatment of LN.

### Data sharing statement

Data not shown in the manuscript include correlation analysis of several urinary cell types with the respective disease activity scores, comparisons of urinary CD3/CD14 and CD3/CD19 ratios between SLE, DN and AAV and expression of CD28 on CD4+ and CD8+ T cells. These datasets are available via email to the corresponding author.

## Additional file

Additional file 1: Table S1. Detailed characteristics of acute lupus nephritis patients. Every row represents one patient. IV (+ V), Lupus nephritis class IV (+ V); G, global involvement; A, active lesions; C, chronic lesions; *pauci immune glomerulonephritis. Aza, azathioprine; Cyc, pulse cyclophosphamide; HCQ, hydroxychloroquine; MMF, mycophenolate mofetil; Pred, prednisolone.

## Abbreviations

AAV: anti-neutrophil cytoplasmatic antibody associated vasculitis
ANCA: anti-neutrophil cytoplasmatic antibody
APC: allophycocyanin, fluorescent dye
AUC: area under the curve
Aza: azathioprine
Belim: belimumab
BSA: bovine serum albumin
BVAS: Birmingham vasculitis activity score
CCR: CC-motif chemokine receptor
CD: cluster of differentiation
Cy5, Cy7: cyanine fluorescent dyes
Cyc: cyclophosphamide
DN: diabetic nephropathy
DRFZ: German Rheumatism Research Center
FITC: fluorescein isothiocyanate, fluorescent dye
Flebo: flebogamma
HCQ: hydroxychloroquine
LN: lupus nephritis
MMF: mycophenolate mofetil
MTX: methotrexate
na: not applicable
PBS: phophate-buffered saline
PE: phycoerythrin, fluorescent dye
PerCP: peridinin-chlorophyll proteins, fluorescent dye
Pred: prednisolone
ROC: receiver-operator characteristic
Rtx: rituximab
SLE: systemic lupus erythematosus
SLEDAI: systemic lupus erythematosus disease activity index
