# Supplementary figures and images for: The cellular signature of urinary immune cells in Lupus nephritis: new insights into potential biomarkers
Source: Arthritis Res Ther. 2015 Apr 3;17(1):94. doi: 10.1186/s13075-015-0600-y (PMC4412098; doi:10.1186/s13075-015-0600-y)

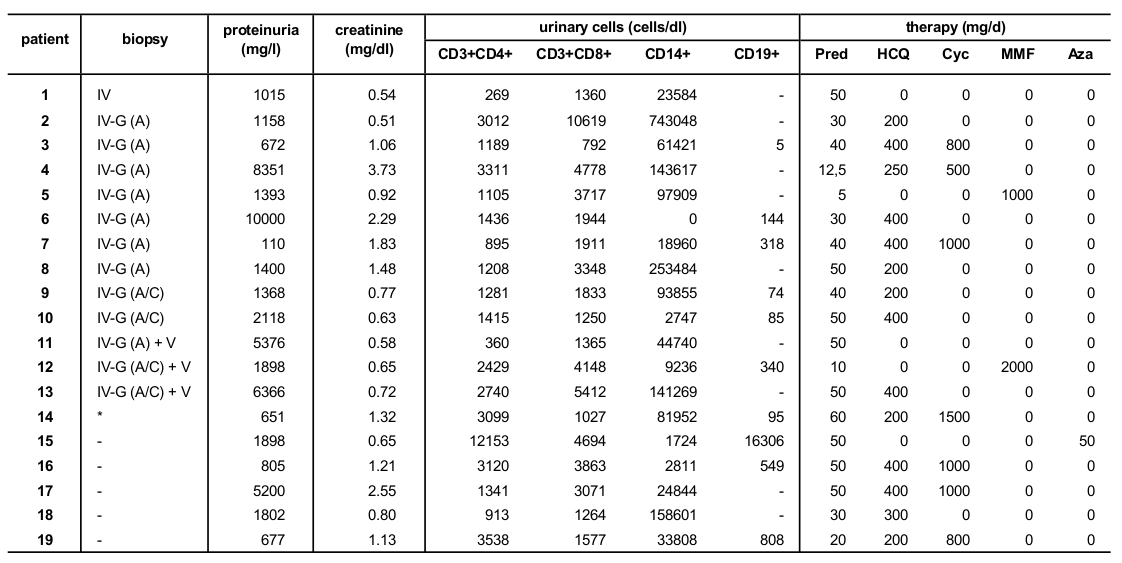

Supplement: Additional file 1: Table S1. — Detailed characteristics of acute lupus nephritis patients. Every row represents one patient. IV (+ V), Lupus nephritis class IV (+ V); G, global involvement; A, active lesions; C, chronic lesions; *pauci immune glomerulonephritis. Aza, azathioprine; Cyc, pulse cyclophosphamide; HCQ, hydroxychloroquine; MMF, mycophenolate mofetil; Pred, prednisolone. [file 13075_2015_600_MOESM1_ESM.png]
